# *Bosea rubneri* sp. nov. Isolated from Organically Grown *Allium cepa*

**DOI:** 10.1007/s00284-024-03717-6

**Published:** 2024-06-05

**Authors:** Dominic A. Stoll, Christina Grimmler, Birgit Hetzer, Alexandra Masoura, Sabine E. Kulling, Melanie Huch

**Affiliations:** 1 Department of Safety and Quality of Fruit and Vegetables, Max Rubner-Institut, Federal Research Institute of Nutrition and Food, Haid-und-Neu-Straße 9, 76131 Karlsruhe, Germany; 2 Department of Safety and Quality of Meat, Max Rubner-Institut, Federal Research Institute of Nutrition and Food, E.-C.-Baumann-Straße 20, 95326 Kulmbach, Germany; 3 Department of Food Technology and Bioprocess Engineering, Max Rubner-Institut, Federal Research Institute of Nutrition and Food, Haid-und-Neu-Straße 9, 76131 Karlsruhe, Germany

## Abstract

Strain ZW T0_25^T^ was isolated from an onion sample (*Allium cepa* var. Hytech F1) within a storage trial and proofed to be a novel, aerobic, Gram-stain negative, rod-shaped bacterial strain. Analyses of the 16S rRNA gene sequence and of the whole draft genome sequences, i.e., digital DNA–DNA hybridization (dDDH), Average Nucleotide Identity (ANI) and Average Amino Acid Identity (AAI) showed that this strain represents a new species of the genus *Bosea*. The genome size of strain ZW T0_25^T^ is 6.19 Mbp, and the GC content is 66.9%. As whole cell sugars, rhamnose, ribose and glucose were identified. Ubiquinone Q-10 is the major respiratory quinone with 97.8%. Polar lipids in strain ZW T0_25^T^ are composed of one phosphatidylethanolamine, one phosphatidylglycerol, one aminophospholipid, two aminolipids, one glycolipid and two phospholipids whereas the fatty acid profile predominantly consists of C18:1 w7c (63.3%), C16:1 w7c (19.5%) and C16:0 (7.1%). Phenotypic traits were tested in the wet lab as well as predicted in silico from genome data. Therefore, according to this polyphasic approach, the new name *Bosea rubneri* sp. nov. with the type strain ZW T0_25^T^ (= DSM 116094^ T^ = LMG 33093^ T^) is proposed.

**Supplementary Information:**

The online version contains supplementary material available at 10.1007/s00284-024-03717-6.

## Introduction

The genus *Bosea* was proposed in 1996 for a new bacterial strain that was isolated from agricultural soil around Calcutta, India [1] and named in honour of J.C. Bose, the founder of the Bose Institute, where the type strain of the type species was isolated [1]. The genus *Bosea* belongs to the alphaproteobacteria family *Boseaceae* [2] and at time of writing, the genus comprises 12 species, (I) *B. caraganae* [3], (II) *B. eneae* [4], (III) *B. lathyri* [5], (IV) *B. lupini* [5], (V) *B. massiliensis* [4], (VI) *B. minatitlanensis* [6], (VII) *B. psychrotolerans* [7], (VIII) *B. robiniae* [5], (IX) *B. spartocytisi* [8], (X) *B. thiooxidans* [1], (XI) *B. vaviloviae* [9] and (XII) *B. vestrisii* [4]. The type strains of half of the 12 species of *Bosea* were isolated from root nodules of different plants belonging to the family Fabaceae [3, 5, 8, 9]. Four type strains were isolated from fresh water, i.e., three of them from hospital water supply [4] and one from Lake Michigan [7]. In addition, one type strain was isolated from an anaerobic sludge blanket reactor fed with wastewater [6] whereas the type strain of the type species [1] was isolated from soil as mentioned above. Strain ZW T0_25^T^ was isolated from an onion sample in November 2020 alongside a collection of 316 bacterial strains within a study with the objective to investigate the bacterial microbiota of different onion varieties. Among this bacterial collection, a number of 13 strains including ZW T0_25^T^ could not be identified by 16S rRNA gene sequencing or MALDI-TOF analysis. However, whole-genome shotgun sequencing of these 13 bacterial strains revealed that these strains including strain ZW T0_25^T^ represent potential new species of different genera. One of these strains, ZW T2_19^T^ has been previously described as *Rathayibacter rubneri* sp. nov. [10].

## Materials and Methods

### Isolation of Bacterial Strain ZW T0_25^T^, Culture Conditions and Morphology

Strain ZW T0_25^T^ was isolated from an onion (*Allium cepa* var. Hytech F1) which was harvested, air-dried and selected for storage at 2 °C. The isolation was performed as published previously [10]. Briefly, about a quarter of an onion bulb was minced, serially diluted in quarter-strength Ringer’s solution, plated on Standard Nutrient I agar (Merck KGaA, Darmstadt, Germany) and incubated at 30 °C. Strains were streaked out until purity and cryopreserved with 15% glycerol. Mid-exponential to stationary phase cells were visualized under a phase-contrast microscope (Leica), and motility was tested with hanging-drop method. In addition, cell morphology was investigated by scanning electron microscopy (SEM) as described previously [11]. For SEM, bacterial cells were grown on Standard Nutrient I agar for 48 h. Furthermore, the cell diameters and cell lengths of 50 cells of strain ZW T0_25^T^ were measured using ObjectJ in ImageJ 1.53q [12].

### Biochemical Characteristics

Growth was tested at 7, 24, 37, and 42 °C in Standard Nutrient I broth as well as at 24 °C, 100 rpm in Standard Nutrient I broth supplemented with 1, 2, 3, 4, 5, 6.5, 8, and 10% NaCl and 0.01, 0.02, or 0.03% potassium tellurite. In addition, growth was tested at pH 5, pH 6, pH 7, pH 8 and pH 9 according to [13] at 24 °C, 100 rpm and in MRS broth supplemented with 1% ox bile. Haemolysis was investigated on blood agar (bioMérieux, Nürtingen, Germany). The presence of catalase was tested according to standard microbiological methods as described in [4, 14], while presence of cytochrome oxidase was tested using Bactident Oxidase test strips (Merck KGaA) according to the instructions of the manufacturer. Biochemical characteristics of strain ZW T0_25^T^ were determined using API 20NE, API 50CH, API Coryne, API ZYM (bioMérieux) at 24 °C according to the manufacturer’s instructions. Chemotaxonomic analyses of strain ZW T0_25^T^ were carried out by the Identification Service of the DSMZ (German Collection of Microorganisms and Cell Cultures, Braunschweig, Germany) including analyses of respiratory quinones [15, 16], fatty acids [15, 17–21], whole cell sugars [22, 23] and polar lipids [24, 25]. For this purpose, the biomass of 10 L liquid culture (Standard Nutrient I broth, 24 °C, 110 rpm, 24 h) of strain ZW T0_25^T^ was collected by centrifugation (9,622 × *g*; 10 min; 4 °C) and the total moist biomass of strain ZW T0_25^T^ (6.06 g) was sent on dry ice to the DSMZ.

### 16S rRNA Gene Sequencing, Genome Sequencing and Analyses

For 16S rRNA gene sequencing, isolation of bacterial DNA of strain ZW T0_25^T^, 16S rRNA gene amplification and analysis were carried out as described previously [26]. Briefly, the bacterial DNA was isolated using the Blood and Tissue Kit (Qiagen), and the almost complete 16S rRNA gene was amplified using the primers 16Sseq fw (5’-ATA GTT TGA TCM TGG CTC AG-3’) and 16Sseq rev (5’-GGN TAC CTT GTT ACG ACT TC-3’). The accession number for the 16S rRNA gene sequence of strain ZW T0_25^T^ at GenBank/EMBL/DDBJ is OR512845. Phylogenetic trees (maximum likelihood, maximum parsimony and neighbour joining) of strain ZW T0_25^T^ and type strains of the genus *Bosea* were built using BioNumerics (version 8.1, Applied Maths). The 16S rRNA gene sequences of the type strains were obtained from NCBI (https://www.ncbi.nlm.nih.gov). In addition, pairwise nucleotide similarities of the 16S rRNA gene sequences were calculated using EZBioCloud [27]. The whole draft genome sequence of strain ZW T0_25^T^ was sequenced on an Illumina MiSeq (Illumina Nextera XT DNA Library Prep Kit, 250 bp paired-end reads) by our group. The reads were assembled de novo using SPAdes genome assembler v. 3.13.1. [28]. This whole-genome shotgun project has been deposited at DDBJ/ENA/GenBank under the accession JAWDID000000000. The version described in this paper is version JAWDID010000000. In addition, digital DNA–DNA hybridization (dDDH) was calculated by using Type (Strain) Genome Server (TYGS) [29, 30], average nucleotide identity (ANI) was calculated as OrthoANIu values by using EzBioCloud [27, 31], and AAI (Average Amino Acid Identity) was determined by using AAI calculator [32]. A Genome BLAST Distance Phylogeny (GBDP) tree (whole-genome sequence based) of strain ZW T0_25^T^ and type strains of *Bosea* was calculated by using TYGS [29]. The software package Traitar [33] and the bioinformatic tool Protologger [34] running on the galaxy platform [35] were used for phenotype and protologue prediction from the genome data, respectively. ResFinder (version 4.1) [36–38] was used for prediction of antimicrobial resistances.

## Results and Discussion

### Morphology

**Fig. 1:**
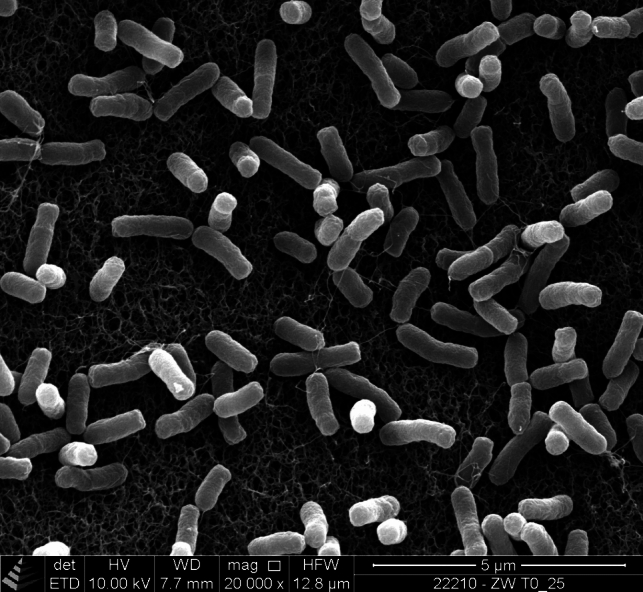
Scanning electron micrograph of cells of strain ZW T0_25^T^. Bacterial cells were grown on Standard Nutrient I agar for 48 h. Bars, 5 μm

After 48 h growth at 30 °C on Standard Nutrient I agar, strain ZW T0_25^T^ occurred as smooth, round and cream-coloured colonies with a diameter of about 4 mm. Strain ZW T0_25^T^ occurred as motile, small rods under the phase-contrast microscope. The rod-shaped morphology was confirmed by SEM (Fig. 1). The mean diameter and mean cell length of strain ZW T0_25^T^ were 0.40 ± 0.05 µm and 1.16 ± 0.23 µm, respectively. This corresponds well to the cell width of 0.4 to 0.85 µm and cell length of 1.1 to 3 µm as reported for other type strains of the genus *Bosea* [1, 3, 5–7, 9].

### Phylogenetic and Genome Analyses

**Fig. 2:**
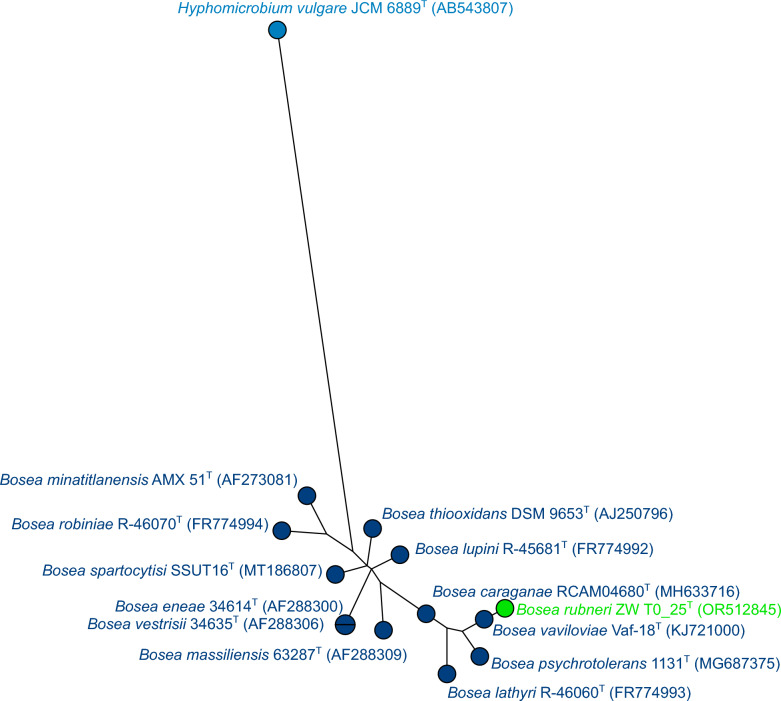
Analysis of 16S rRNA gene sequences of strain ZW T0_25^T^ and all twelve type strains of *Bosea. Hyphomicrobium vulgare* JCM 6889^T^ was used as an outgroup. The tree was built using maximum likelihood with Jukes–Cantor as the evolutionary model (BioNumerics, version 8.1; Applied Maths). Square-root scaling was used to display branch length

The 16S rRNA gene sequence (1059 bp, Genbank accession number OR512845) of strain ZW T0_25^T^ was used for BLASTn search and was classified as belonging to the genus *Bosea*. Figure 2 shows a maximum-likelihood tree based on the 16S rRNA gene sequences of all type strains of *Bosea* and shows clearly that strain ZW T0_25^T^ belongs to the genus *Bosea* but clusters apart from the other type strains. The cluster analysis was repeated with two additional clustering methods, namely neighbour joining and maximum parsimony (Figs. S1 and S2), and confirmed that strain ZW T0_25^T^ belongs to the genus *Bosea* with a similarity value of 97.99% to the nearest neighbours according to 16S phylogeny, i.e., the type strains of *B. eneae* and *B. vestrisii* (Fig. S1). Pairwise nucleotide similarity comparisons of the 16S rRNA gene sequences using EZBioCloud [27] of strain ZW T0_25^T^ to the type strains of all *Bosea* species showed that approximately half of the values are below the threshold value for species delimitation of 98.7% [39] (Table S1). Despite the other half had values above the threshold, this also happens in the pairwise comparisons of the type strains of other *Bosea* species (Table S1). Another example where this commonly happens is the genus *Bradyrhizobium* [40]. In any case, the threshold value of 16S rRNA gene similarities is only one part of the picture for a new species description and should be accompanied by other criteria like DDH, ANI as well as discriminant phenotypic properties [39].

**Fig. 3:**
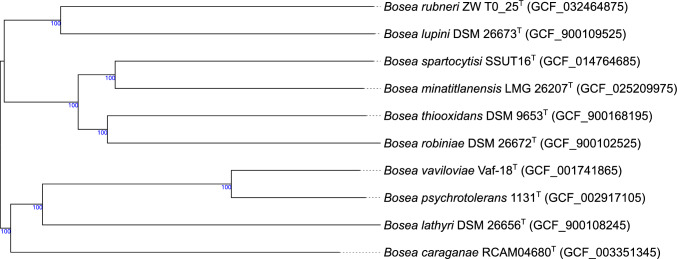
Genome BLAST Distance Phylogeny (GBDP) tree (whole-genome sequence based) of strain ZW T0_25^T^ and all nine type strains of *Bosea*. The tree was built using the type strain genome server (TYGS) [29]. Tree inferred with FastME 2.1.6.1 [45] from GBDP distances calculated from genome sequences. The branch lengths are scaled in terms of GBDP distance formula d5. The numbers above branches are GBDP pseudo-bootstrap support values > 60% from 100 replications, with an average branch support of 91.3%. The tree was rooted at the midpoint [46]

Characteristics of the genome sequences (e.g., genome size, GC% content and number of proteins) of strain ZW T0_25^T^ and nine type strains of *Bosea* for which the whole genomes were available, were obtained by the TYGS [29] and are summarized in Table 1. The genome characteristics of strain ZW T0_25^T^ were well comparable to genome characteristics of the type strains of *Bosea.* The genome size of ZW T0_25^T^ has a length of 6.19 Mbp, and the genome sizes of *Bosea* type strains range from 4.71 to 6.72 Mbp. The genome of ZW T0_25^T^ shows 66.9 GC%, and *Bosea* type strains show 64.8 to 68.6 GC%. A number of 5910 proteins is predicted for ZW T0_25^T^ and proteins of *Bosea* type strains range from 4507 to 6078 proteins. These characteristics of the genome of ZW T0_25^T^ were confirmed as analysed by Protologger [34]. In addition, the results of dDDH, ANI and AAI values are included in Table 1. The dDDH values of strain ZW T0_25^T^ compared to the type strains of the genus *Bosea* were clearly below the threshold of 70% for species delimitation [41, 42] with the highest similarity of strain ZW T0_25^T^ to the type strain *B. lupini* with a dDDH value of 27.1%. All OrthoANIu values of strain ZW T0_25^T^ compared to the type strains of *Bosea* were below the 95–96% cut-off value for species delimitation [43] and similar to the results of dDDH: The highest similarity compared to strain ZW T0_25^T^ was observed for the type strain *B. lupini* with a value of 83.66%. All AAI values of strain ZW T0_25^T^ compared to the type strains of *Bosea* were below the 95–96% cut-off value for species delimitation as proposed by [44]. Again, the highest similarity compared to strain ZW T0_25^T^ was observed for the type strain *B. lupini* with a value of 81.42%. Comparable to the results of dDDH, ANI and AAI, the GBDP tree of strain ZW T0_25^T^ and type strains of *Bosea* confirmed that the nearest neighbour of strain ZW T0_25^T^ was the type strain of *B. lupini* (Fig. 3).

**Table 1:** Whole draft genome characteristics, values of digital DNA–DNA hybridization (dDDH), average nucleotide identity (ANI) and average amino acid identity (AAI) of ZW T0_25^T^ and type strains of the genus *Bosea*

Characteristic	1	2	3	4	5	6	7	8	9	10
Genome length (Mbp)^#^	6.19	6.17	5.92	6.13	4.71	6.39	5.38	5.92	5.37	6.72
GC%^#^	66.9	66.9	64.8	66.7	68.6	65.8	66.3	66.4	67.4	65.4
Number of proteins^#^	5910	5639	5477	5921	4507	5899	5006	5600	5054	6078
dDDH [%]^#^ versus ZW T0_25^T^	–	24.4	23.4	27.1	23.7	23.6	23.2	23.3	23.8	23.8
OrthoANIu [%]^‡^ versus ZW T0_25^T^	–	81.02	79.69	83.66	80.50	80.14	79.94	79.55	80.41	80.01
AAI [%]^§^ vs ZW T0_25^T^	–	74.56	75.18	81.42	73.78	74.38	74.12	72.70	75.03	74.54

Strain: 1, *B. rubneri* sp. nov. ZW T0_25^T^ (GCF_032464875); 2, *B. caraganae* RCAM04680^T^ (GCF_003351345); 3, *B. lathyri* DSM 26656^ T^ (GCF_900108245); 4, *B. lupini* LMG 26383^ T^ (GCF_900109525); 5, *B. minatitlanensis* LMG 26207^ T^ (GCF_025209975); 6, *B. psychrotolerans *1131^T^ (GCF_002917105); 7, *B. robiniae* DSM 26672^ T^ (GCF_900102525); 8, *B. spartocytisi* SSUT16^T^ (GCF_014764685); 9, *B. thiooxidans* DSM 9653^ T^ (GCF_900168195); 10, *B. vaviloviae* Vaf-18^ T^ (GCF_001741865)

For the species *B. eneae, B. massiliensis* and *B. vestrisii* no genome sequences were available at Genbank

#Values were obtained from TYGS [29, 30]. For dDDH formula d4 is given

‡Values were obtained from EzBioCloud [27, 31]

§Values were obtained from [32]

### In Silico and In Vivo Phenotypical and Biochemical Analyses

The in silico prediction for the phenotype of strain ZW T0_25^T^ as predicted by Traitar [33] was well comparable to the predictions for the other *Bosea* type strains (Table S2). Phenotypic characteristics that are positive for strain ZW T0_25^T^ according to both predictors in Traitar are aerobe, bacillus-similar morphology, Gram-negative and motile as well as catalase and oxidase positive which fits very well to the results of the wet lab analysis. Furthermore, strain ZW T0_25^T^ was predicted to be bile-susceptible, to grow in KCN and to be colistin-polymyxin susceptible. In addition, growth on ordinary blood agar was predicted by Traitar, which was confirmed in the laboratory: Strain ZW T0_25^T^ grows well on blood agar and shows γ-haemolysis. Moreover, the optimal growth conditions in Standard Nutrient I liquid medium for strain ZW T0_25^T^ were observed to be at 24 °C. Furthermore, strain ZW T0_25^T^ did not grow in MRS supplemented with 1% ox bile within 7 days which agrees with the Traitar prediction. In addition, in Traitar no growth was predicted in presence of 6.5% NaCl which was confirmed in the wet lab as growth was tested at 1 up to 10% NaCl and strain ZW T0_25^T^ did only grow up to 1% NaCl. Growth of ZW T0_25^T^ was observed at pH 7 and pH 8. No growth was observed in Standard Nutrient I at 42 °C nor in Standard Nutrient I supplemented with 0.01, 0.02 or 0.03% potassium tellurite at 24 °C. However, growth was observed in Standard Nutrient I without supplements at 7 °C after 7 days which is comparable to *B. psychrotolerans* which grows at temperatures higher than 5 °C [7]. In the API 20NE test, ZW T0_25^T^ was positive for the assimilation of potassium gluconate and weakly positive for the assimilation of adipic acid. The assimilation of potassium gluconate has also been described for the type strains of *B. spartocytisi, B. lupini, B. lathyri, B. robiniae, B. eneae, B. massilie*nsis and *B. vestrisii* as well as for the type strain of the type species *B. thiooxidans* [4, 7, 8]. Assimilation of adipic acid is also described for the type strains of *B. thiooxidans, B. massiliensis, B. vestrisii, B. eneae, B. lupini* and *B. lathyri* [4, 5]. Strain ZW T0_25^T^ did not ferment any of the substrates (carbohydrates and derivatives) tested with API 50CH. This is in good agreement with the literature as *B. minatitlanensis, B. eneae, B. masssiliensis, B. vestrisii* and *B. thiooxidans* are also described to give negative results of all substrates tested with API 50CH [4, 6]. According to API Coryne and API ZYM, the following enzymatic activities were detected: pyrrolidonyl arylamidase, alkaline phosphatase, esterase, esterase lipase, acid phosphatase, leucine arylamidase and naphtol-AS-BI-phosphohydrolase. In addition, acetate production from acetyl-CoA, propionate production from propanoyl-CoA, L-cysteine and acetate production from sulphide and L-serine, as well as L-glutamate production from ammonia were predicted by Protologger. According to ResFinder and Protologger, no antibiotic resistance was detected in the genome of strain ZW T0_25^T^.

### Analyses of Respiratory Quinones, Fatty Acids, Whole Cell Sugars and Polar Lipids

For strain ZW T0_25^T^, ubiquinone Q-10 was detected as the major quinone via HPLC–DAD and confirmed by mass spectrometry with 97.8%. In addition, minor amounts of Q-9 and Q-11 were detected with 0.7 and 1.6%, respectively. To the best of our knowledge, respiratory quinone data are only available for the type strains of *B. thiooxidans* [1], *B. psychrotolerans, B. vaviloviae* and *B. lathyri* [7]. A predominance of Q-10 with more than 97% has been detected in those four species of *Bosea* and in addition in ZW T0_25^T^. Furthermore, minor amounts of Q-11 are present in all *Bosea* species. In addition, Q-9 has been detected in ZW T0_25^T^ in minor amounts (0.7%) which is described for the first time to be present in a species belonging to the genus *Bosea*. The polar lipid profile of strain ZW T0_25^T^ was composed of one phosphatidylethanolamine, one phosphatidylglycerol, one aminophospholipid, two aminolipids, one glycolipid and two phospholipids (Fig. S3). To the best of our knowledge, polar lipids were described only for the type species *B. thiooxidans* [1] and for *B. psychrotolerans, B. vaviloviae* and *B. lathyri* [7]*.* Phosphatidylethanolamine and phosphatidylglycerol have been described for the abovementioned species of *Bosea* and are also present in ZW T0_25^T^. In addition, two aminolipids and one unidentified glycolipid have been detected in ZW T0_25^T^ and are also described for *B. vaviloviae* and *B. lathyri*. The most noticeable difference in the polar lipid profile of strain ZW T0_25^T^ is the absence of phosphatidylcholine, diphosphatidylglycerol and phosphatidylmonoethylamine compared to the other species of *Bosea*. The fatty acid profile of strain ZW T0_25^T^ predominantly consisted of C18:1 w7c (63.3%), C16:1 w7c (19.5%) and C16:0 (7.1%) (Table S3). A high occurrence of C18:1 w7c was already stated as a feature in the description of the genus *Bosea* [1] which has been reported for all other type strains of *Bosea* species so far [3–9]. It is noticeable that no presence of C19:0 cyclo w8c was detected in strain ZW T0_25^T^ as this feature can be found in all other type strains of *Bosea*. In addition, the analysis of the whole-cell hydrolysate of strain ZW T0_25^T^ showed the presence of rhamnose, ribose and glucose as major whole cell sugars with traces of mannose.

## Conclusion

In this study, a polyphasic approach including genomic as well as phenotypic and biochemical analysis was used to investigate the taxonomical position of strain ZW T0_25^T^. The genomic analysis comprised *i.a*. 16S rRNA gene comparisons, dDDH, ANI and AAI and phylogenomic clustering with closely related type strains of the genus *Bosea*. The results of this study indicate that strain ZW T0_25^T^ represents the type strain of a novel species within the genus *Bosea*, for which the name *Bosea rubneri* sp. nov. is proposed.

### Description of *Bosea rubneri* sp. nov.

*Bosea rubneri* sp. nov. (rub´ne.ri. N.L. gen. n. *rubneri*, of Rubner, referring to Max Rubner, a German physiologist after whom the Max Rubner-Institute was named, and where the type strain was isolated).

Cells occur as motile, Gram-stain negative and small, single rods. Mean size of cells is 1.16 µm in length and 0.40 µm in width. Colonies (diameter ≈ 4 mm) occur smooth, round and cream coloured after 48 h on Standard Nutrient I agar at 30 °C. Oxidase positive and catalase positive. Growth was observed at temperatures between 7 and 37 °C. The optimal growth temperature is 24 °C. Growth occurs in the presence of up to 1% NaCl and at pH 7 and pH 8 at 24 °C. No gas formation in Standard Nutrient I broth. It shows γ-haemolysis on blood agar. Cells are capable of producing pyrrolidonylarylamidase, alkaline phosphatase, esterase, esterase lipase, leucine arylamidase, acid phosphatase, naphthol-AS-BI-phosphohydrolase and β-glucosidase. Cells were able to assimilate L-arabinose, potassium gluconate and adipic acid. No other positive reactions are observed using API 20NE, API 50CH, API Coryne and API ZYM. The major respiratory quinone is Q-10. Rhamnose, ribose and glucose are detected as major whole cell sugars. The polar lipids are one phosphatidylethanolamine, one phosphatidylglycerol, one aminophospholipid, two aminolipids, one glycolipid and two phospholipids.

The type strain ZW T0_25^T^ was isolated from a bulb of an onion hybrid race (*Allium cepa* var. Hytech F1) grown in Kleinhohenheim (Germany). Strain ZW T0_25^T^ has been deposited as DSM 116094^T^ and LMG 33098^T^. The GenBank/EMBL/DDBJ accession number for the 16S rRNA gene sequences of strain ZW T0_25^T^ is OR512845. The whole-genome shotgun project of strain ZW T0_25^T^ has been deposited at DDBJ/ENA/GenBank under the accession JAWDID000000000. The version described in this paper is version JAWDID010000000. The genome size of the type strain is 6.19 Mbp and the GC content is 66.9%.

### Supplementary Information

Below is the link to the electronic supplementary material.

Supplementary file1 (PDF 463 KB)

## Declarations

### Research Involving Human and/or Animal Participants

This article does not contain any studies with animals or human participants.
