# Supplementary material for: Bosea rubneri sp. nov. Isolated from Organically Grown Allium cepa
Source: Curr Microbiol. 2024 Jun 5;81(7):212. doi: 10.1007/s00284-024-03717-6 (PMC11153308; doi:10.1007/s00284-024-03717-6)
Supplement: Supplementary file 1 — Supplementary file1 (PDF 463 KB) [file 284_2024_3717_MOESM1_ESM.pdf]

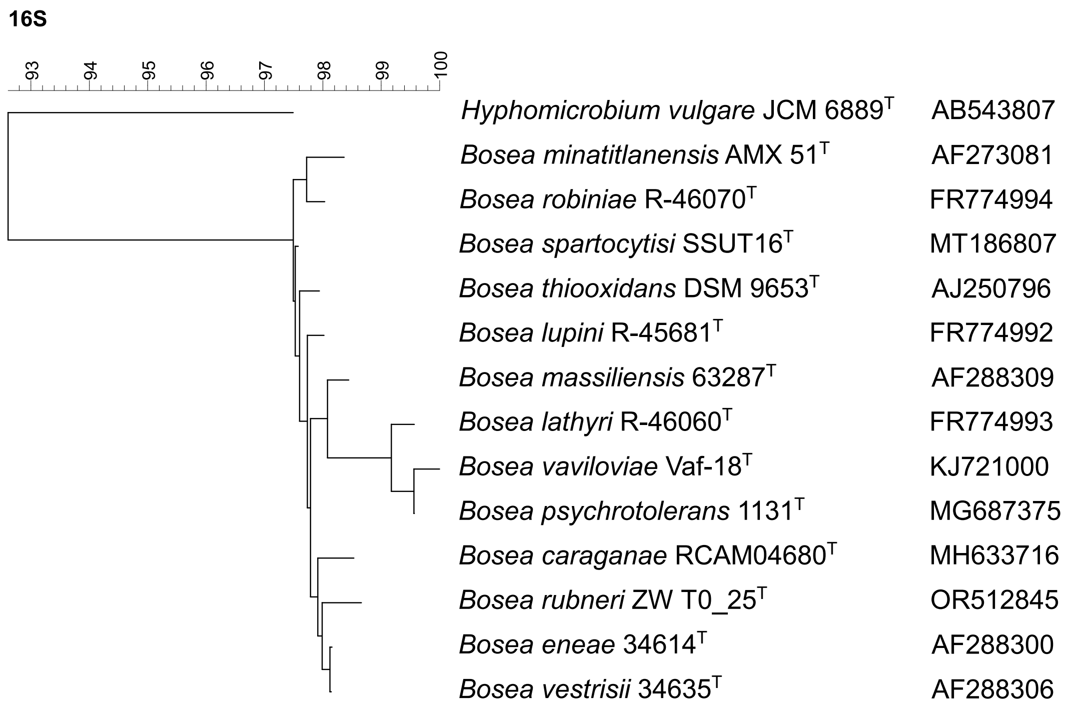


**Fig. S1.** Analysis of 16S rRNA gene sequences of strain ZW T0_25$^{T}$ and all 12 type strains of *Bosea. Hyphomicrobium vulgare* JCM 6889$^{T}$ was used as an outgroup. Clustering was performed using fast algorithm and neighbour joining (BioNumerics, version 8.1; Applied Maths).

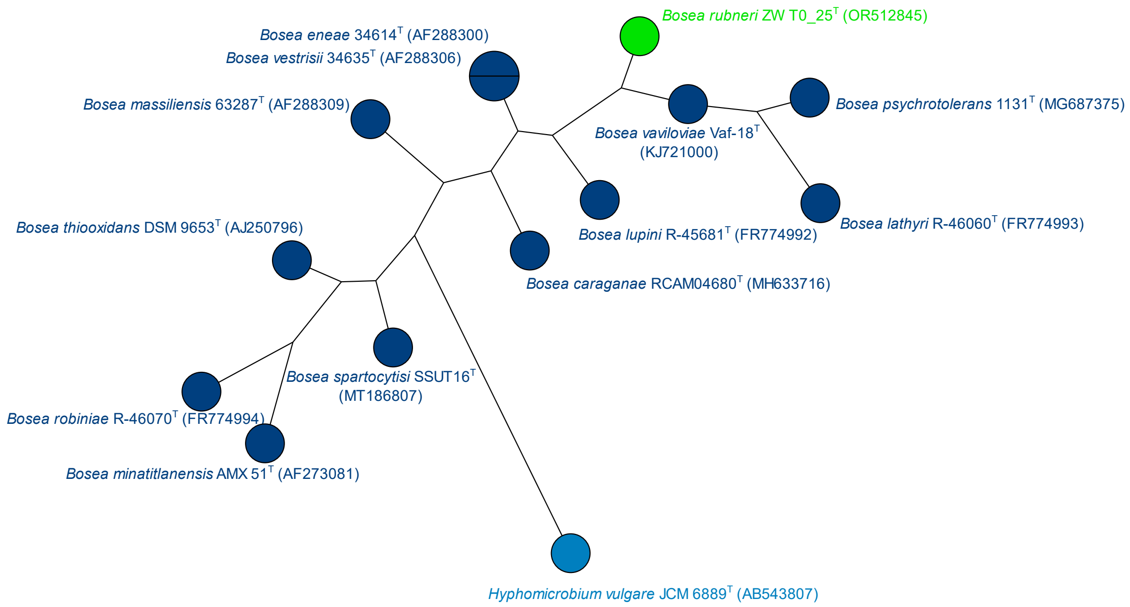


**Fig. S2.** Analysis of 16S rRNA gene sequences (length ca. 1500 bp) of strain ZW T0_25$^T$ and all 12 type strains of *Bosea. Hyphomicrobium vulgare* JCM 6889$^T$ was used as an outgroup. The tree was built using maximum parsimony (BioNumerics, version 8.1; Applied Maths). Square root scaling was used to display branch length.

**Table S1. Pairwise nucleotide similarities of the 16S rRNA gene sequences of ZW T0_25$^T$ versus type strains of the genus *Bosea*$^*$.**
Strain: 1, *B. rubneri* sp. nov. ZW T0_25$^T$ (OR512845); 2, *B. caraganae* RCAM04680$^T$ (MH633716); 3, *B. eneae* 34614$^T$ (AF288300); 4, *B. lathyri* R-46060T$^T$ (FR774993); 5, *B. lupini* R-45681$^T$ (FR774992); 6, *B. massiliensis* 63287$^T$ (AF288309); 7, *B. minatitlanensis* AMX51$^T$ (AF273081); 8, *B. psychrotolerans* 1131$^T$ (MG687375); 9, *B. robiniae* R-46070$^T$ (FR774994); 10, *B. spartocytisi* SSUT16$^T$ (MT186807); 11, *B. thiooxidans* DSM 9653$^T$ (AJ250796); 12, *B. vaviloviae* Vaf-18$^T$ (KJ721000); 13, *B. vestrisii* 34635$^T$ (AF288306)

| Pairwise Nucleotide Similarity [%]* | 1 | 2 | 3 | 4 | 5 | 6 | 7 | 8 | 9 | 10 | 11 | 12 | 13 |
|---|---|---|---|---|---|---|---|---|---|---|---|---|---|
| 1 | 100 | 98.68 | 99.15 | 98.77 | 99.05 | 98.49 | 97.45 | 99.34 | 98.02 | 98.75 | 98.58 | 99.62 | 99.15 |
| 2 | - | 100 | 99.07 | 98.93 | 98.86 | 98.85 | 98.07 | 98.74 | 98.21 | 98.76 | 98.43 | 98.64 | 99.07 |
| 3 | - | - | 100 | 98.25 | 99.58 | 98.88 | 97.99 | 98.39 | 98.81 | 99.20 | 99.03 | 98.43 | 99.93 |
| 4 | - | - | - | 100 | 98.12 | 98.23 | 97.57 | 99.12 | 97.71 | 97.96 | 97.78 | 98.86 | 98.18 |
| 5 | - | - | - | - | 100 | 99.01 | 98.54 | 98.32 | 99.03 | 99.56 | 99.45 | 98.36 | 99.58 |
| 6 | - | - | - | - | - | 100 | 98.32 | 98.32 | 98.44 | 98.90 | 98.67 | 98.35 | 98.88 |
| 7 | - | - | - | - | - | - | 100 | 97.51 | 99.03 | 98.98 | 98.84 | 97.43 | 98.07 |
| 8 | - | - | - | - | - | - | - | 100 | 97.80 | 98.18 | 97.95 | 99.56 | 98.39 |
| 9 | - | - | - | - | - | - | - | - | 100 | 99.42 | 98.96 | 97.72 | 98.81 |
| 10 | - | - | - | - | - | - | - | - | - | 100 | 99.49 | 98.47 | 99.27 |
| 11 | - | - | - | - | - | - | - | - | - | - | 100 | 97.93 | 99.03 |
| 12 | - | - | - | - | - | - | - | - | - | - | - | 100 | 98.43 |
| 13 | - | - | - | - | - | - | - | - | - | - | - | - | 100 |

Values were obtained using EZBioCloud [27].

*Pairwise nucleotide similarities above the threshold value of 98.7% [39] are coloured in grey.

**Table S2. Phenotypes predicted by Traitar (https://galaxy.bifo.helmholtz-hzi.de/) for strain ZW T0_25$^T$ and of other nine type strains of the genus *Bosea***, for which genome sequences were available. 0, no predictor positive; 1, one predictor positive and labelled in light blue; 2, two predictors positive and labelled in dark blue.

| Category | Phenotype | *Bosea rubneri* sp. nov. ZW T0_25$^T$ | *B. thiooxidans* DSM 9653$^T$ | *B. robiniae* DSM 26672$^T$ | *B. minatitlanensis* LMG 26207$^T$ | *B. lupini* DSM 26673$^T$ | *B. caraganae* RCAM04680$^T$ | *B. spartocytisi* SSUT 16$^T$ | *B.lathyri* DSM 26656$^T$ | *B. vaviloviae* VAF_18$^T$ | *B. psychrotolerans* 1131$^T$ |
|---|---|---|---|---|---|---|---|---|---|---|---|
| enzyme | Pyrrolidonyl--naphthylamide | 0 | 0 | 0 | 0 | 0 | 1 | 1 | 2 | 0 | 0 |
| enzyme | Beta-hemolysis | 0 | 0 | 0 | 0 | 0 | 0 | 0 | 0 | 0 | 0 |
| enzyme | Nitrite to gas | 0 | 0 | 0 | 0 | 0 | 0 | 0 | 0 | 0 | 0 |
| enzyme | Coagulase production | 1 | 1 | 1 | 1 | 0 | 1 | 0 | 1 | 0 | 0 |
| enzyme | Nitrate-to-nitrite conversion | 2 | 2 | 1 | 1 | 2 | 2 | 1 | 2 | 2 | 2 |
| enzyme | Alkaline phosphatase | 2 | 2 | 2 | 2 | 2 | 2 | 2 | 2 | 2 | 2 |
| enzyme | Lipase | 2 | 2 | 2 | 2 | 1 | 2 | 2 | 2 | 2 | 2 |
| growth | Growth at 42°C | 0 | 0 | 0 | 1 | 0 | 0 | 0 | 0 | 0 | 1 |
| growth | DNase | 0 | 0 | 0 | 0 | 0 | 0 | 0 | 0 | 0 | 0 |
| growth | Growth in 6.5% NaCl | 0 | 0 | 0 | 0 | 0 | 0 | 0 | 0 | 0 | 0 |
| growth | Mucate utilization | 1 | 1 | 1 | 1 | 1 | 1 | 1 | 1 | 1 | 1 |
| growth | Growth on MacConkey agar | 1 | 2 | 1 | 2 | 1 | 2 | 2 | 1 | 1 | 1 |
| growth | Bile susceptible | 2 | 2 | 2 | 2 | 2 | 2 | 2 | 2 | 2 | 2 |
| growth | Growth in KCN | 2 | 2 | 2 | 2 | 2 | 2 | 2 | 2 | 2 | 2 |
| growth | Colistin-polymyxin susceptible | 2 | 2 | 2 | 2 | 2 | 2 | 2 | 2 | 2 | 2 |
| growth | Growth on ordinary blood agar | 2 | 2 | 2 | 2 | 2 | 2 | 2 | 2 | 2 | 2 |
| growth amino acid | Indole | 0 | 0 | 0 | 0 | 0 | 0 | 0 | 0 | 0 | 0 |
| growth amino acid | Ornithine decarboxylase | 0 | 0 | 0 | 0 | 0 | 0 | 0 | 0 | 0 | 0 |
| growth amino acid | Lysine decarboxylase | 0 | 0 | 0 | 0 | 0 | 0 | 0 | 0 | 0 | 0 |
| growth amino acid | Arginine dihydrolase | 2 | 0 | 0 | 2 | 0 | 0 | 0 | 2 | 0 | 2 |
| growth carboxylic acid | Citrate | 0 | 0 | 0 | 0 | 0 | 0 | 0 | 0 | 0 | 0 |
| growth carboxylic acid | Tartrate utilization | 0 | 0 | 0 | 1 | 1 | 1 | 1 | 1 | 1 | 1 |
| growth carboxylic acid | acetate utilization | 2 | 1 | 1 | 0 | 2 | 1 | 1 | 2 | 0 | 2 |

| | | | | | | | | | | | |
|---|---|---|---|---|---|---|---|---|---|---|---|
| growth carboxylic acid | Malonate | 1 | 1 | 1 | 1 | 1 | 2 | 1 | 1 | 1 | 1 |
| growth glucose | Glucose fermenter | 0 | 0 | 0 | 0 | 0 | 0 | 0 | 0 | 0 | 0 |
| growth glucose | Methyl red | 0 | 0 | 0 | 0 | 0 | 0 | 0 | 0 | 0 | 0 |
| growth glucose | Voges-Proskauer | 0 | 0 | 0 | 0 | 0 | 0 | 0 | 0 | 0 | 0 |
| growth glucose | Gas from glucose | 0 | 0 | 0 | 0 | 0 | 0 | 0 | 0 | 0 | 0 |
| growth glucose | Glucose oxidizer | 2 | 2 | 2 | 2 | 2 | 2 | 2 | 2 | 2 | 2 |
| growth sugar | Maltose | 0 | 0 | 0 | 0 | 0 | 0 | 1 | 0 | 1 | 0 |
| growth sugar | D-Mannose | 0 | 0 | 0 | 0 | 0 | 0 | 0 | 0 | 0 | 1 |
| growth sugar | D-Sorbitol | 0 | 0 | 0 | 0 | 0 | 0 | 0 | 0 | 0 | 0 |
| growth sugar | ONPGc (-galactosidase) | 0 | 0 | 0 | 0 | 0 | 0 | 0 | 0 | 0 | 0 |
| growth sugar | Lactose | 0 | 0 | 0 | 0 | 0 | 0 | 0 | 0 | 0 | 0 |
| growth sugar | Raffinose | 0 | 0 | 0 | 0 | 0 | 0 | 0 | 0 | 0 | 0 |
| growth sugar | L-Rhamnose | 0 | 0 | 0 | 0 | 0 | 0 | 0 | 0 | 0 | 0 |
| growth sugar | Sucrose | 0 | 0 | 0 | 0 | 0 | 0 | 0 | 0 | 0 | 0 |
| growth sugar | Salicin | 0 | 0 | 0 | 0 | 0 | 0 | 0 | 0 | 0 | 0 |
| growth sugar | Melibiose | 0 | 0 | 0 | 0 | 0 | 0 | 0 | 0 | 0 | 0 |
| growth sugar | Starch hydrolysis | 0 | 0 | 0 | 0 | 0 | 0 | 0 | 0 | 0 | 0 |
| growth sugar | D-Mannitol | 1 | 1 | 1 | 1 | 1 | 1 | 1 | 1 | 1 | 1 |
| growth sugar | Trehalose | 1 | 0 | 2 | 0 | 2 | 1 | 1 | 1 | 1 | 2 |
| growth sugar | Cellobiose | 1 | 1 | 1 | 1 | 1 | 2 | 1 | 0 | 0 | 0 |
| growth sugar | L-Arabinose | 1 | 0 | 0 | 0 | 1 | 1 | 1 | 1 | 1 | 1 |
| growth sugar | Glycerol | 2 | 2 | 2 | 2 | 2 | 2 | 2 | 2 | 0 | 0 |
| growth sugar | D-Xylose | 1 | 1 | 1 | 2 | 2 | 2 | 2 | 1 | 2 | 2 |
| growth sugar | Urea hydrolysis | 2 | 2 | 2 | 2 | 2 | 2 | 2 | 2 | 2 | 2 |
| growth sugar | myo-Inositol | 2 | 2 | 2 | 1 | 2 | 1 | 1 | 2 | 1 | 1 |
| growth sugar | Esculin hydrolysis | 1 | 1 | 2 | 1 | 2 | 1 | 2 | 2 | 2 | 2 |
| morphology | Yellow pigment | 0 | 0 | 0 | 0 | 0 | 0 | 0 | 0 | 0 | 1 |
| morphology | Gram positive | 0 | 0 | 0 | 0 | 0 | 0 | 0 | 0 | 0 | 0 |
| morphology | Spore formation | 0 | 0 | 0 | 0 | 0 | 0 | 0 | 0 | 0 | 0 |
| morphology | Coccus—clusters or groups predominate | 0 | 0 | 0 | 0 | 0 | 0 | 0 | 0 | 0 | 0 |
| morphology | Coccus | 0 | 0 | 0 | 0 | 0 | 0 | 0 | 0 | 0 | 0 |

| | | | | | | | | | | | |
|---|---|---|---|---|---|---|---|---|---|---|---|
| morphology | Coccus—pairs or chains predominate | 0 | 0 | 0 | 0 | 0 | 0 | 0 | 0 | 0 | 0 |
| morphology | Bacillus or coccobacillus | 2 | 2 | 2 | 2 | 2 | 2 | 2 | 2 | 2 | 2 |
| morphology | Gram negative | 2 | 2 | 2 | 2 | 2 | 2 | 2 | 2 | 2 | 2 |
| morphology | Motile | 2 | 2 | 2 | 2 | 2 | 2 | 2 | 2 | 2 | 2 |
| oxygen | Anaerobe | 0 | 0 | 0 | 0 | 0 | 0 | 0 | 0 | 0 | 0 |
| oxygen | Facultative | 0 | 0 | 0 | 0 | 0 | 0 | 0 | 0 | 0 | 0 |
| oxygen | Capnophilic | 0 | 1 | 1 | 1 | 1 | 1 | 1 | 1 | 0 | 1 |
| oxygen | aerobe | 2 | 2 | 2 | 1 | 2 | 2 | 2 | 2 | 2 | 2 |
| oxygen enzyme | Oxidase | 2 | 2 | 2 | 2 | 2 | 2 | 0 | 0 | 0 | 2 |
| oxygen enzyme | Catalase | 2 | 2 | 2 | 2 | 2 | 2 | 2 | 2 | 2 | 2 |
| product | Hydrogen sulfide | 0 | 0 | 0 | 0 | 0 | 0 | 0 | 0 | 0 | 0 |
| proteolysis | Gelatin hydrolysis | 0 | 0 | 0 | 0 | 0 | 0 | 0 | 0 | 0 | 0 |
| proteolysis | Casein hydrolysis | 0 | 0 | 0 | 0 | 1 | 2 | 1 | 1 | 2 | 2 |

## DSMZ Identification Services polar lipid report

PE = Phosphatidylethanolamine

PG = Phosphatidylglycerol

APL = Aminophospholipid

AL = Aminolipid

GL = Glycolipid

PL = Phospholipid

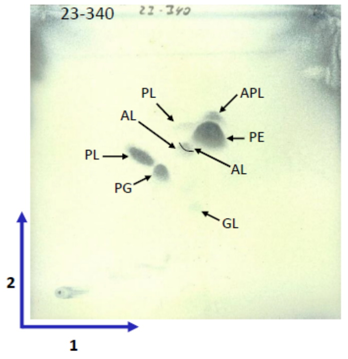


Fig. S3. Polar lipid report as provided by DSMZ Identification Services for strain ZW T0_25$^T$.

**Table S3. Fatty acid composition of *B. rubneri* sp. nov. ZW T0_25T.**

| Fatty acids* | Percent |
|---|---|
| $C_{14:0}$ | 0.2 |
| $C_{14:0}$ DMA | 0.1 |
| $C_{15:0}$ | 0.3 |
| $C_{16:0}$ | 7.1 |
| $C_{16:0}$ 3OH | 3.5 |
| $C_{16:1}$ w5c | 2.7 |
| $C_{16:1}$ w7c | 19.5 |
| $C_{17:0}$ | 0.4 |
| $C_{17:1}$ w6c | 0.2 |
| $C_{17:1}$ w8c | 0.6 |
| $C_{18:0}$ | 0.3 |
| $C_{18:1}$ w7c | 63.3 |
| $C_{18:1}$ w7c 11-methyl | 1.4 |
| $C_{18:1}$ w9c | 0.5 |

* Analyses were carried out by the Identification Service of the DSMZ (German Collection of Microorganisms and Cell Cultures, Braunschweig, Germany).
